# LC-MS/MS Analysis Unravels Deep Oxidation of Manganese Superoxide Dismutase in Kidney Cancer

**DOI:** 10.3390/ijms18020319

**Published:** 2017-02-04

**Authors:** Zuohui Zhao, Kazem M. Azadzoi, Han-Pil Choi, Ruirui Jing, Xin Lu, Cuiling Li, Fengqin Wang, Jiaju Lu, Jing-Hua Yang

**Affiliations:** 1 Cancer Research Center, Shandong University School of Medicine, Jinan 250012, China; zhaozuohui@126.com (Z.Z.); jingruirui223@163.com (R.J.); lvxin121@163.com (X.L.); cuilingli@yahoo.com (C.L.); wfqlzq@163.com (F.W.); 2 Departments of Urology and Pediatric Surgery, Shandong Provincial Hospital Qianfoshan Hospital, Shandong University, No. 324, Jingwu Road, Jinan 250021, China; 3 Departments of Surgery, Urology, Pathology and Proteomics Laboratory, Veterans Affairs Boston Healthcare System, Boston University School of Medicine, 150 S. Huntington Ave., Boston, MA 02130, USA; kazadzoi@bu.edu (K.M.A.); hpchoi89@gmail.com (H.-P.C.)

**Keywords:** liquid chromatography-tandem mass spectrometry, MNSOD, oxidation, ROS, quantitative proteomics

## Abstract

Manganese superoxide dismutase (MNSOD) is one of the major scavengers of reactive oxygen species (ROS) in mitochondria with pivotal regulatory role in ischemic disorders, inflammation and cancer. Here we report oxidative modification of MNSOD in human renal cell carcinoma (RCC) by the shotgun method using data-dependent liquid chromatography tandem mass spectrometry (LC-MS/MS). While 5816 and 5571 proteins were identified in cancer and adjacent tissues, respectively, 208 proteins were found to be up- or down-regulated (*p* < 0.05). Ontological category, interaction network and Western blotting suggested a close correlation between RCC-mediated proteins and oxidoreductases such as MNSOD. Markedly, oxidative modifications of MNSOD were identified at histidine (H^54^ and H^55^), tyrosine (Y^58^), tryptophan (W^147^, W^149^, W^205^ and W^210^) and asparagine (N^206^ and N^209^) residues additional to methionine. These oxidative insults were located at three hotspots near the hydrophobic pocket of the manganese binding site, of which the oxidation of Y^58^, W^147^ and W^149^ was up-regulated around three folds and the oxidation of H^54^ and H^55^ was detected in the cancer tissues only (*p* < 0.05). When normalized to MNSOD expression levels, relative MNSOD enzymatic activity was decreased in cancer tissues, suggesting impairment of MNSOD enzymatic activity in kidney cancer due to modifications. Thus, LC-MS/MS analysis revealed multiple oxidative modifications of MNSOD at different amino acid residues that might mediate the regulation of the superoxide radicals, mitochondrial ROS scavenging and MNSOD activity in kidney cancer.

## 1. Introduction

Superoxide dismutases (SODs) are the ubiquitous superfamily of antioxidant metalloenzymes that convert the superoxide anion (O_2_^−^) into oxygen and hydrogen peroxide (H_2_O_2_) when cells are exposed to oxidation. These enzymes are the major scavengers of reactive oxygen species (ROS), the natural side products during the process of aerobic cells respiration in physiological and pathological conditions [1]. Three forms of superoxide dismutases, namely Cu/ZnSOD (SOD1/3 in humans), Fe/MNSOD (also called MNSOD, SOD2 in humans), and NiSOD (only found in bacteria), are identified [2]. Among them, MNSOD is the primary mitochondrial ROS scavenging enzyme that catalyzes the conversion of superoxide to H_2_O_2_, which is subsequently transformed to water by catalase and other peroxidases [1,3]. MNSOD is essential for the survival of all aerobic organisms from bacteria to humans under physiological conditions [1,4].

Posttranslational modifications (PTMs) play a major role in regulating MNSOD activities, interactions, and localization [1]. Protein oxidative modification, a major class of PTM, is caused by oxidative or nitrative disruption of amino acid residues. Oxidative protein modification has been described during aging and various pathological conditions, and serves as a useful biomarker for assessing oxidative stress processes in aging and disease conditions [4]. Protein oxidation can lead to hydroxylation of aromatic groups and aliphatic amino acid side chains, nitration of aromatic amino acid residues, sulfoxidation of methionine residues [5]. Some specific enzymes, such as tyrosine hydroxylase (TH), facilitate the oxidation process in vivo [6]. Aromatic amino acid side chains, such as tyrosine (Tyr, Y) and tryptophan (Trp, W), are more susceptible to free radical incursion leading to specific modification of the aromatic ring. For example, tyrosine (Tyr) yields greater 3,4-dihydroxyphenylalanine (DOPA) and less 2,4-isomer [7]. Protein oxidation can lead to diverse functional consequences, such as loss of enzymatic and binding activities, increased susceptibility to aggregation, proteolysis and altered immunogenicity [8]. Overall, it is believed that protein oxidation is central to functional deficit of the target proteins [9].

In addition to direct oxidative damage, cells could undergo nitrative damage through reactive nitrogen species (RNS) pathway, which often occurs with the redox-sensitive amino acid residues including tyrosine and tryptophan [10]. Nitration on tyrosine 58 (Y^58^ or Y^34^ when the 24 amino acids transit peptide is cleaved) of MNSOD has been detected by crystal structures and mutation analysis [1,11]. Previous data showed that human MNSOD Y^58^ was exclusively nitrated to 3-nitrotyrosine (3-NT) to inactivate its enzymatic activity, and nitration of Y^58^ alone was sufficient for inactivation of MNSOD enzymatic activity [1]. Similar to nitration, acetylation of MNSOD was shown to impair its enzymatic activity. Recent evidence suggests the presence of multiple MNSOD acetylation sites on lysine (Lys, K), such as 53, 68, 89, 122, 130 [12]. It was also shown that SIRT3 could deacetylate MNSOD and enhance its enzymatic activity in vitro [1,3,13].

Oxidative stress in mitochondria becomes a major source of ROS. Free radicals originating from mitochondria interact with surrounding molecules and initiate a cascade of signaling pathways leading to oxidative modifications of cellular organelles. Oxidative modifications within mitochondrial proteins may contribute to the development of carcinogenesis [14]. The precise mechanisms by which oxidative and nitrative conditions affect MNSOD structure and function remain unclear. While MNSOD is reportedly up-regulated in kidney cancer [15,16], its enzymatic activity in tumor areas is similar to adjacent kidney tissues [16,17], suggesting that MNSOD enzymatic activity does not necessarily correlate with its protein expression. It was recently shown that oxidative stress-mediated MNSOD modifications compromised its enzymatic activity and led to functional deficit [1,3,18,19]. In this study, we first pinpointed MNSOD as a vital protein in the kidney tissues from clear cell renal cell carcinoma (ccRCC) using liquid chromatography-tandem mass spectrometry (LC-MS/MS), then attempted to identify and quantify crucial sites of oxidative modifications of MNSOD using high resolution LC-MS/MS and Progenesis LC-MS software, respectively. The newly identified (to the best of our knowledge) oxidation sites we report may introduce novel perspectives for the refinement of MNSOD regulatory mechanisms. Characterization of MNSOD oxidative modifications may be vital for interpretation of previous studies on MNSOD in tumor biology and could enhance our intervening ability in tumorigenesis.

## 2. Results

### 2.1. Quantitative Proteomic Analysis Suggests the Involvement of Anti-Oxidative Stress Pathway in ccRCC

**Figure 1:**
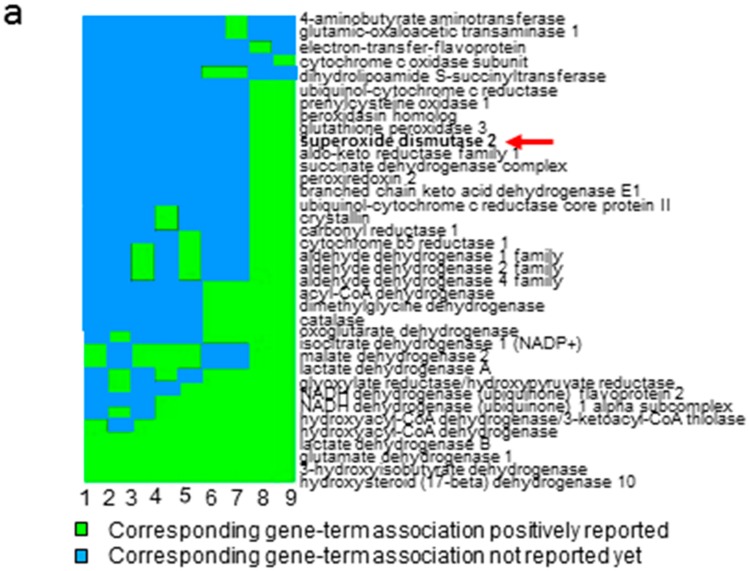

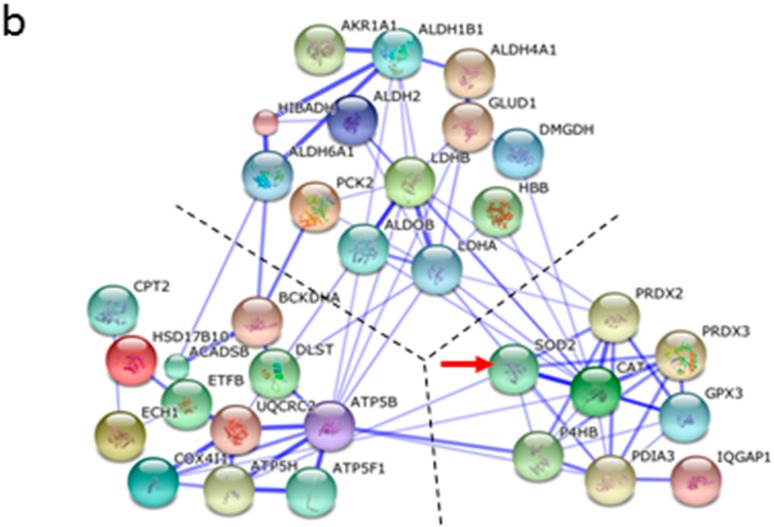
Quantitative proteomic analysis revealed the importance of anti-oxidative stress pathway in ccRCC. (**a**) The heatmap showed the 33 oxidoreductases (by DAVID) were involved in binding cofactor, coenzyme, NAD, NADH or NAD(P). 1: NAD binding site; 2: NAD or NADH binding; 3: nucleotide phosphate-binding region; 4: NAD(P)-binding domain; 5: NAD; 6: coenzyme binding; 7: cofactor binding; 8: oxidation reduction; 9: oxidoreductase. Red arrow showed the candidate protein (MNSOD, SOD2). Green area: gene-term association positively reported, light blue area: gene term association not reported yet; (**b**) visualization of protein–protein interactions of the 37 oxidation-reduction related proteins in ccRCC using STRING analysis (confidence mode). 37 oxidation-reduction related proteins were input into STRING software and they formed three main clusters (only 33 connected proteins were shown and the clusters were divided by dotted lines), among which MNSOD (SOD2, red arrow) were participated in the network and were chosen to be validated later. The solid lines represented interactions between proteins and thickness of the solid lines denoted the confidence level associated with each interactions.

Three pairs of tumor and adjacent tissues from ccRCC patients were analyzed by LC-MS/MS. Totally, 5571 and 5816 non-redundant proteins with FDR < 0.01 were identified in adjacent and ccRCC tissues, respectively (Table S1). Among the identified proteins, the average spectral counts of three repeats were used to evaluate their expression levels, which revealed 100 up- and 108 down-regulated proteins for at least 1.4 folds in ccRCC vs. adjacent tissues with the criteria of *p* < 0.05, detected in every replicate, and the average spectral counts of >20 (Table S2). According to Database for Annotation, Visualization, and Integrated Discovery (DAVID) functional annotation, 33 of the 208 dysregulated proteins were related to oxido-reductases (*p* = 2.87 × 10^−18^) (Tables S3 and S4). Heatmap analysis (Figure 1a) denoted the involvement of these oxidoreductases in binding with cofactors, coenzyme, NAD, NADH and/or NAD(P). Notably, MNSOD was among these oxidoreductases, suggesting that mitochondrial MNSOD was also involved in the electron transport chain for ROS removal. Consistent with these observations, ontological category based on biological process using Search Tool for the Retrieval of Interacting Genes/Proteins (STRING) (Table S5) indicated that one of the most significant categories was oxidation-reduction process (37 proteins, *p* = 6.49 × 10^−17^) (Table S6). Three main interactive clusters were formed among the 37 interacting proteins with MNSOD as an important node (Figure 1b). Because cancer cells usually demand high ROS concentrations to maintain their high proliferation rate [14], these data suggested that oxido-reductases, particularly MNSOD, played an important role in RCC pathogenesis [16].

### 2.2. Oxidative Modification of MNSOD

**Figure 2:**
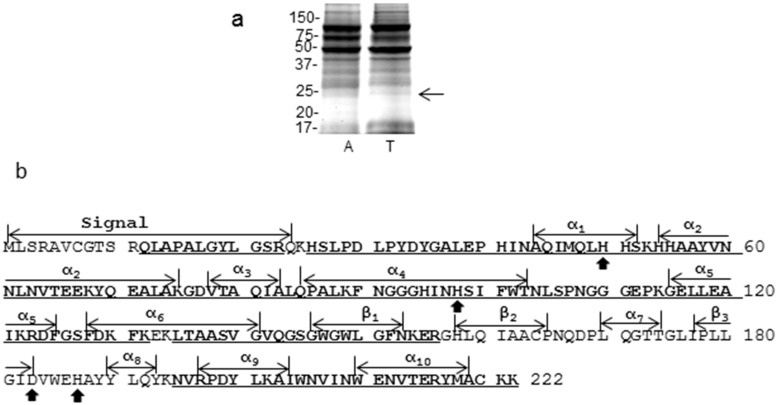
LC-MS/MS coverage of MNSOD. (**a**) Whole cell lysates from kidney tissues were separated by SDS-PAGE and stained by Coomassie Blue (arrow indicated MNSOD). The gel image is the representative of 4 pairs of tumor and adjacent tissues used for PTMs analysis. A: adjacent; T: tumor; (**b**) CID-based sequence coverage of MNSOD. After Coomassie Blue staining, the 22 kDa protein bands corresponding to MNSOD were cut from the gel and digested, and peptides were analyzed by LC-MS/MS on LTQ-Orbitrap mass spectrometer (MS). The underlined amino acids (bold letters) were identified by Proteome Discoverer 1.4 (MASCOT and SEQUEST), which covered 76.13% sequence of MNSOD. Signal: the signal peptide; α: the α-helices; β: the β-sheets, subscript numbers represent original numbers; solid arrows: metal (Mn^2+^) binding sites.

For a deep post-translational modification analysis, MNSOD was excised from SDS-PAGE (Figure 2a) and analyzed by LC-MS/MS. With a standard search using MASCOT and SEQUEST, 18 high confident peptides of MNSOD were identified, which covered 76% of the sequence (Figure 2b).

Next, the MS/MS data were searched again with an open modification search algorithm [20] to identify the peptides of MNSOD containing oxidation at any possible amino acids with a delta mass of +16 Da (Table 1). Totally 168 of +16 modification events were counted, 81 of which occurred at tryptophan (W), 27 at glycine (G), 24 at tyrosine (Y), 14 at histidine (H), 13 at asparagine (N), 7 at alanine (A), and 2 at valine (V).

To confirm whether these +16 modifications were due to oxidation, restricted search with dynamic oxidation (+15.995 Da) was performed using MASCOT and SEQUEST. High confident oxidation modifications were confirmed at tryptophan (W), tyrosine (Y), histidine (H), and asparagine (N) residues of MNSOD (Table 2). Markedly, these oxidation sites were located in three hotspots, including hotspot 1 (H^54^, H^55^, Y^58^), hotspot 2 (W^147^, W^149^) and hotspot 3 (W^205^, N^206^, N^209^, W^210^). Additionally, the known nitration (+44.985 Da) at tyrosine (Y^58^) was also identified in the kidney tissues (Table 2).

### 2.3. Tryptophan Oxidation at W^147^/W^149^ and W^205^/W^210^ of MNSOD

Over half of the observed oxidation events of MNSOD occurred at tryptophan. Four potentially oxidized sites, W^147^/W^149^ and W^205^/W^210^, were identified in two hotspots near the C-terminus. In the hotspot around the residues from 200 to 212 (hotspot 3), two of the oxidized tryptophan residues were found in the peptide AIW^205^NVINW^210^ENVTER. The oxidized W^210^ (Figure S1a) was identified by the unmodified b_4–7_ ions and the mass shift of the b_8–13_ ions, supported by the unmodified y_2–6_ and the modified y_7–12_ ions, which has been reported before [21]. Similarly, oxidation at W^205^ (Figure S1b) was identified by the unmodified y_3–9,11_ ions followed by the modified y_12_ ion; it was supported by the shifted b_5,7–8,10–11_ ions. The other oxidized tryptophan residues were found in the peptide LTAASVGVQGSGW^147^GW^149^LGFNK in hotspot 2. The oxidized tryptophan at W^147^ (Figure S1c) was uncovered by the unmodified y_4–5,7_ and modified y_8–12,14_ ions, which was supported by the unmodified b_6,8–9,11_ and modified b_16,19_ ions. Double oxidation at W^147^ and W^149^ in a different MS/MS profile of the same peptide (Figure S1d) was identified by the unmodified b_6,8–9,11_ ions and double mass shifts of 15.99 Da in the b_15–19_ ions. Markedly, these identified tryptophan oxidations at W^147^, W^149^, W^205^ and W^210^ were located in the hotspots that were reported to be important for the enzymatic activity of MNSOD [2]. Notably, tryptophan hydroxylase (TPH), a well characterized enzyme in mammals, was shown to convert tryptophan to 5-hydroxytryptophan, the precursor for the neurotransmitter serotonin and melatonin [22], although it was unclear whether TPH was involved in tryptophan oxidation in proteins.

### 2.4. Asparagine Oxidation at N^206^ and N^209^ of MNSOD

The asparagine residue in proteins might be oxidized to form beta-hydroxyl asparagine [23]. We identified two asparagine oxidation sites at N^206^ and N^209^ in MNSOD, and the two oxidized asparagine residues were found in the peptide AIWN^206^VIN^209^WENVTER, which was located in the C-terminal. Notably, this modification was observed only in combination with other modification: double oxidation at W^205^N^206^ and N^209^W^210^ in the same peptide (Table 2). The oxidized N^206^ in the peptide (Figure S1e) was identified by the unmodified y_3–10_ ions and the double mass shifts of 15.99 Da in the y_12_ ion, and it was further supported by the double mass shifts of 15.99 Da in the ions after b_4_. The oxidized N^209^ (Figure S1f) was identified by the first shift between the b_4–6_ ions and b_7_ ion, and the oxidized W^210^ identified by the second shift of 15.99 Da between the b_7_ ion and b_8–13_ ions; the double oxidized asparagine/tryptophan residues were confirmed by the unmodified y_3–6_ ions and double mass shifts of 15.99 Da in the y_8–12_ ions. Therefore, we concluded that the asparagine residues at N^206^ and N^209^ were hotspots for oxidation that could be spontaneously oxidized synchronously with tryptophan.

### 2.5. Histidine Oxidation at H^54^ and H^55^ of MNSOD

Histidine oxidations in proteins are commonly found in cells undergoing redox-mediated oxidation of histidine to 2-oxo-histidine. The oxidized product has been suggested as a biological marker for oxidatively modified proteins [24]. In this study, we identified two oxidized histidine residues at H^54^ and H^55^ in MNSOD. The oxidized histidine at H^54^ in the peptide H(54)HAAYVNNLNVTEEK (Figure S1g) was identified by the mass shifts at b_2–14_ ions, which was supported by the unmodified y_2–14_ ions followed by the total mass shift. The double oxidized histidine at H^54^ and H^55^ in the peptide H^54^H^55^AAYVNNLNVTEEK (Figure S1h) was identified by double mass shifts at b_11,13_ ions, which was supported by the unmodified y_4,6–13_ ions followed by 32 Da shift of the total mass. The two oxidative histidine residues were located in the first hotspot for oxidation, which was also reported previously in human medulloblastoma cells [21].

### 2.6. Tyrosine Oxidation at Y^58^ of MNSOD

Tyrosine could be oxidized to form 3-hydroxyl tyrosine or 3,4-dihydroxyphenylalanine (L-DOPA) by tyrosine hydroxylase (TH) [6,10]; however, it was unclear whether TH oxidizes the tyrosine residue in proteins. The oxidized tyrosine at Y^58^ was identified because the mass shift of 15.99 Da was absent in the y_4–10_ ions, but present in the y_11–14_ ions in the MS/MS profile of the peptide HHAAY^58^VNNLNVTEEK (Figure S1i); it was further confirmed by the absence of the extra mass in the b_2,4_ ions and presence in the b_5–14_ ions.

### 2.7. Oxidation at H^54^, H^55^, Y^58^, W^147^ and W^149^ of MNSOD Is Up-Regulated in ccRCC

**Figure 3:**
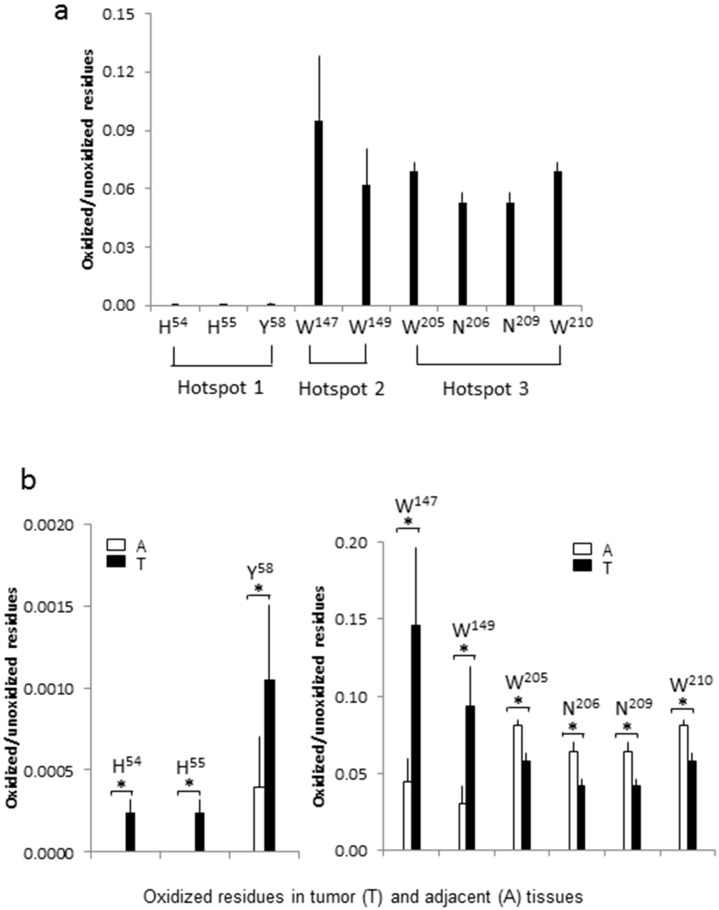
Quantification of the oxidized residues of MNSOD. (**a**) The average ratios of the oxidized to unoxidized residues in tumor + adjacent tissues. Abundances of the modified and unmodified peptide fragments containing the nine oxidized and unoxidized residues (H^54^, H^55^, Y^58^, W^147^, W^149^, W^205^, N^206^, N^209^ and W^210^) were used to calculate the ratios (Table S7). Three hotspots are indicated; (**b**) comparison of the oxidation modification between tumor and adjacent tissues. The relative ratios of oxidized to unoxidized residues at histidine 54 (H^54^), 55 (H^55^) and tyrosine 58 (Y^58^) were low both in ccRCC tumor tissues and adjacent tissues but, dramatically increased in tumor comparing with adjacent tissues (the left panel, * *p* < 0.03, *n* = 4) (Table S7). The relative ratios at tryptophan 147 (W^147^) and 149 (W^149^) were also increased significantly in tumor, but the relative ratios at tryptophan 205 (W^205^), asparagine 206 (N^206^), asparagine 209 (N^209^) and tryptophan 210 (W^210^) were decreased slightly in tumor (the right panel, * *p* < 0.03, *n* = 4) (Table S7).

The peak areas were commonly considered proportional to the amounts of the peptide. In this study, the level of an oxidized residue was measured by the areas of all peptide fragments containing the oxidized residue. In comparison with unoxidized, the relative level of oxidized residues at H^54^, H^55^, Y^58^, W^147^, W^149^, W^205^, N^206^, N^209^ and W^210^ were calculated as 0.00012, 0.00012, 0.00073, 0.09530, 0.06171, 0.06938, 0.05298, 0.05298 and 0.06938, respectively, in kidney tissues (Figure 3a, Table S7). Apparently, the basal levels of oxidation in MNSOD might indicate a managed balance between formation and neutralization of ROS in vivo. Note that abundances of W^205^ and N^206^ were identical to W^210^ and N^209^ because they were from two identical peptides with the same abundance but different pattern. The abundances of H^54^ and H^55^ were similar because they came from a low abundant, single oxidation peptide (H^54^) and a high abundant, double oxidation peptide (H^54^ and H^55^), so that their total abundances were mainly contributed by the high abundant peptide. Markedly, oxidation at H^54^ and H^55^ only found in kidney cancer tissues (*p* < 0.05, *n* = 4), and oxidation at Y^58^, W^147^ and W^149^ increased 2.63, 3.26 and 3.13-fold (*p* < 0.05, *n* = 4) in kidney cancer tissues, respectively, in comparison with those in adjacent non-cancer tissues of the same patient (Figure 3b). While, oxidation at N^206^ and N^209^ decreased 1.54-fold (*p* < 0.05, *n* = 4) and oxidation at W^205^ and W^210^ decreased 1.40-fold (*p* < 0.05, *n* = 4) in kidney cancer tissues. Thus, these data suggested that oxidation at certain amino acid positions of MNSOD could be dysregulated in kidney cancer.

### 2.8. The Relative MNSOD Enzymatic Activity Is Attenuated in ccRCC

**Figure 4:**
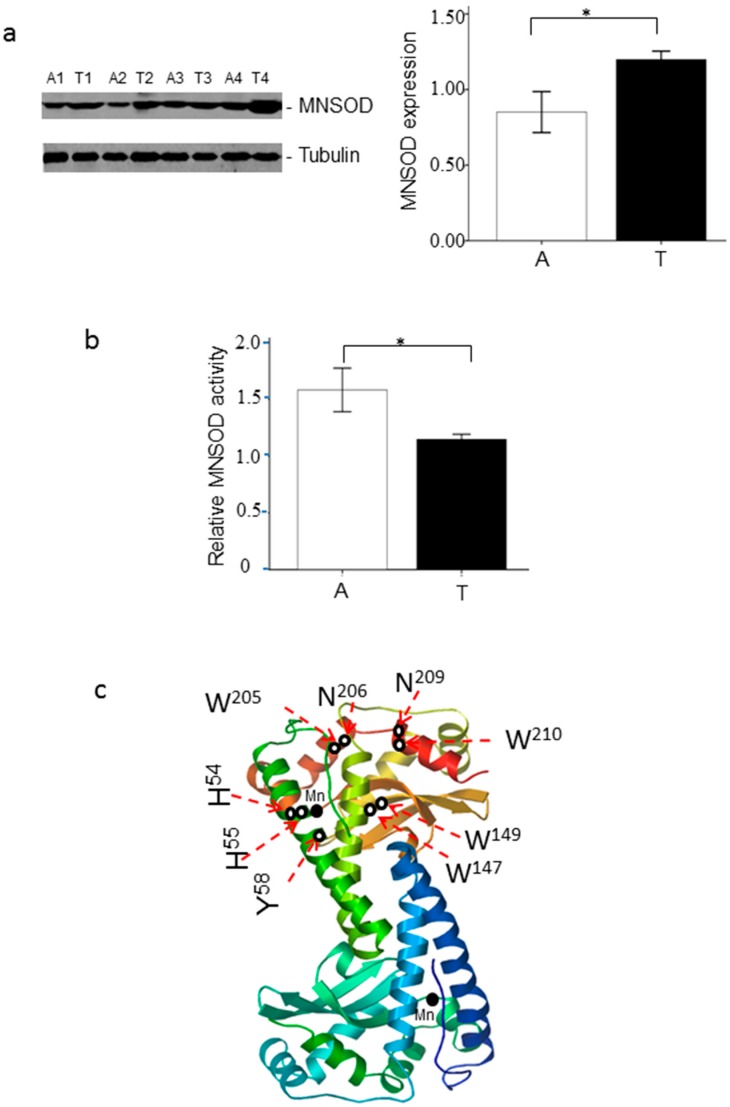
MNSOD expression, enzymatic activity and the potential oxidation hotspots of MNSOD. (**a**) The expression of MNSOD in adjacent (A) and ccRCC (T) tissues. Four individual ccRCC and adjacent tissue lysates were separated by SDS-PAGE and the expression of MNSOD was detected by Western blotting. Tubulin served as the loading control (* *p* < 0.05, *n* = 4); (**b**) MNSOD enzymatic activity in adjacent (A) and ccRCC (T) tissues. Superoxide dismutase (SOD) activity kit was used to measure MNSOD enzymatic activity. The relative MNSOD enzymatic activity was normalized by MNSOD expression (* *p* < 0.05, *n* = 4); (**c**) the potential oxidation hotspots in three dimensional structure of MNSOD. The ribbon structure of MNSOD subunit (1LUV.pdb) is depicted with the two separate polypeptide chains. The active sites (manganese ions, Mn) are depicted as solid spheres and the oxidative modification sites (H^54^, H^55^, Y^58^, W^147^, W^149^, W^205^, N^206^, N^209^, W^210^) are indicated as red dashed arrows. The 3D structure shows the three oxidation hotspots (hotspot 1 (H^54^, H^55^, Y^58^), hotspot 2 (W^147^, W^149^) and hotspot 3 (W^205^, N^206^, N^209^, W^210^)) are distributed in the surface of homotetrameric MNSOD and point to protein internal, which composite a hydrophobic side chain and affect the molecular structure of MNSOD. The colors in the ribbon structure of MNSOD subunit are the automatically displayed colors according to 1LUV.pdb.

Western blotting analysis of four individual ccRCC and adjacent tissue lysates showed a significant increase of MNSOD expression in ccRCC (*p* < 0.05, Figure 4a). Then MNSOD enzymatic activity was measured from ccRCC and adjacent kidney tissue lysates using superoxide dismutase (SOD) activity kit. When normalized by MNSOD expression level, the relative enzymatic activity of MNSOD was decreased in ccRCC in comparison with adjacent tissues (*p* < 0.05, Figure 4b), although the total activity was similar. This observation suggested that modification of MNSOD might impair its enzymatic activity.

## 3. Discussion

MNSOD is highly expressed in kidney and is reportedly up-regulated in ccRCC [15,16], the most common subtype of RCC in adults. Label-free quantitative proteomics approach, which is based on the number of acquired spectra for each protein or the ion intensities of identical peptides, has become a popular alternative to assess relative amount of peptides or proteins [25]. In this study, label-free quantification was performed to compare a number of proteins in kidney tissues, and 208 proteins were found to meet the criteria for dysregulation between ccRCC and adjacent tissues. Among them, MNSOD, a crucial protein regulating ROS was pinpointed as an important target by STRING and DAVID bioinformatics tools. It was followed by ion intensity-based quantification to compare oxidized peptides of MNSOD between ccRCC and adjacent tissues. Using MNSOD protein bands from 4 pairs of kidney samples, shotgun proteomics, and open search algorithm, this approach revealed 9 oxidative modification sites at 4 different amino acid residues on endogenous MNSOD protein (Table 2). Modified sites involved two histidine (H^54^ and H^55^), one tyrosine (Y^58^), four tryptophan (W^147^, W^149^, W^205^ and W^210^) and two asparagine (N^206^ and N^209^), and most of the oxidized residues clustered at the C-terminus. Consistent with our findings, it was recently reported that MNSOD could be oxidized at H^54^, H^55^ and W^210^ in medulloblastoma cells [21]. Notably, three hotspots at amino acid residues 54–58, 147–149, and 205–210 were observed and multiple oxidation incidents occurred simultaneously in the nearby positions, such as W^147^W^149^ and N^209^W^210^. Our data revealed that MNSOD could be oxidized at tryptophan/asparagine residues, while tryptophan oxidation and asparagine hydroxylation were previously reported to alter the structure and/or function of the oxidized protein [26,27]. These observations demonstrated that mitochondrial MNSOD was not only upregulated in kidney cancer, but it was also deeply oxidized at several hotspots.

Based on three-dimensional structure (1LUV.pdb), the catalytic core (active site) of MNSOD is located between the N-terminal helices and the C-terminal α/β domain, coordinating in a strained trigonal bipyramidal geometry by the side chains of His50, His98, Asp183 and His187 and the Mn ion in the active metal ion (Figure 2b and Figure 4c) [3,28]. The residues near the active site were known to affect the enzymatic activity or the stability of sites involving His54, Tyr58 and Trp147 [28,29,30]. The hydrophobic side chains that surrounded the metal-ligand cluster including His51, His54, His55, Tyr58, Phe101, Trp102, Trp149, Tyr190, and Tyr200 could also be altered [31]. Therefore, oxidation modifications of these residues in and around the MNSOD active site were believed to affect the catalytic activity [32]. In this study, we found that the oxidized residues were close to the active site or located in the hydrophobic side chains, which surrounded the manganese-ligand cluster and formed a hydrophobic pocket on the surface of proteins. These included the residues His54 (H^54^), His55 (H^55^), Tyr58 (Y^58^), Trp147 (W^147^) and Trp149 (W^149^) (Figure 4) [2,5,6,9,31]. These observations are supported by previous reports demonstrating that mutations and modifications close to the active site significantly affect the structural stability as well as the catalytic activity of MNSOD [33]. In addition, the relative enzymatic activity, when normalized by MNSOD expression, was decreased in ccRCC in comparison with the adjacent tissues. We, therefore, speculated that the newly identified oxidative modification may initially affect the structure then compromise the catalytic activity of MNSOD, and thereby contribute to ccRCC formation and development.

Using MS, we found that human endogenous mitochondrial MNSOD can undergo oxidative modifications at histidine, tyrosine, tryptophan and asparagine residues. To our knowledge, we report for the first time that human MNSOD can be oxidized at tyrosine and asparagine residues. Although MNSOD oxidation at H^54^, H^55^ and W^210^ was reported in medulloblastoma cells [21], the extent of oxidation and its modification patterns have not been fully elucidated. Asparagine hydroxylation of ankyrin repeat domain (ARD) of ankyrin R by factor-inhibiting hypoxia-inducible factor (FIH) altered the structure and function of the erythrocyte cytoskeletal ankyrin protein [27], and tyrosine oxidation affected mouse bone marrow mesenchymal stem cells (BMMSCs) proliferation and differentiation [26], but little is known about asparagine and tyrosine oxidation in MNSOD. Label-free quantification demonstrated a significant increase of oxidation at H^54^, H^55^, Y^58^, W^147^ and W^149^ in ccRCC, comparing with adjacent tissues (Figure 3b). As protein oxidation usually altered the enzymatic activity [8], we speculated that the oxidation of H^54^, H^55^, Y^58^, W^147^ and W^149^ mediated MNSOD activity, and thereby, contributed to ccRCC initiation. It also accounts for the inconsistency of enzymatic activity and MNSOD expression in ccRCC versus adjacent tissues. Cancer cells produce greater amount of ROS than their corresponding normal cells, thus more MNSOD aggregates in ccRCC to scavenge ROS. However, increased ROS also inhibits MNSOD activity via oxidative mechanisms in ccRCC. Our observations suggest that, in addition to overexpression of MNSOD in cancer cells, MNSOD oxidation might also contribute to ROS regulation by mediation of MNSOD activity [34].

## 4. Materials and Methods

### 4.1. Sample Preparation and Protein Extraction

Kidney cancer tissues (ccRCC) and adjacent morphologically normal kidney cortex (adjacent tissues) were collected from 7 cancer patients with nephrectomy (5 males and 2 females, age from 46 to 75, Fuhrman nuclear grading with 2 G_1_ + 2 G_2_ + 2 G_3_ + 1 G_4_, TNM staging with 2 T_1_ + 3 T_2_ + 2 T_3_) in full compliance with Institutional Ethics Review Board’s guidance. All procedures were consistent with the National Institutes of Health Guide and approved by the institutional board with patients’ written consent. This study was evaluated and approved by the Ethics Committee of Shandong Provincial Hospital Affiliated to Shandong University. Frozen tissues were homogenized in RIPA lysis buffer (Millipore, Billerica, MA, USA) including the protease inhibitor cocktail (Roche, Basel, Switzerland). Protein concentration was determined using BCA protein assay (Biyuntian, Beijing, China). Total proteins of the ccRCC and adjacent tissues from 3 patients (TNM staging with 1 T_1_ + 1 T_2_ + 1 T_3_) were subjected to quantitative proteomic analysis, and those from 4 patients (TNM staging with 1 T_1_ + 2 T_2_ + 1 T_3_) were used for PTMs and MNSOD enzymatic activity analyses.

### 4.2. Liquid Chromatography Tandem Mass Spectrometry

For quantitative proteomic analysis, ~100 μg of total proteins from the tumor and adjacent tissues were separated on sodium dodecyl sulfate polyacrylamide gel electrophoresis (SDS-PAGE), roughly divided into 10 slices according to molecular weights, and digested with trypsin as described previously [35]. The extracted peptides from each gel band were desalted by ZipTip (Millipore) and subjected to peptide fractionation using an EASY-nLC II system (Thermo Scientific, Waltham, MA, USA). The gradient-eluted peptides were analyzed by a Velos Pro ion trap mass spectrometer (Thermo Scientific). The liquid chromatography column (150 mm × Ø 0.075 mm) was packed with 3 μm 100 Å PepMap C18 (Thermo Scientific). Samples were analyzed using 120 min linear gradient of 5%–35% acetonitrile in 0.1% formic acid with a flow rate of 300 nL/min (solvent A: 0.1% formic acid in water, solvent B: 0.1% formic acid in acetonitrile) and the mass spectrometer was operated in a data-dependent mode, in which MS/MS fragmentation was performed using the 20 most intense peaks of every full MS scan. MS/MS spectra were searched against the human protein database (UniProtKB; 88,295 entries) using SEQUEST HT, which is part of the Proteome Discoverer 1.4 data analysis package (Thermo Scientific, San Jose, CA, USA). Trypsin (full cleavage) was specified as cleavage enzyme allowing up to two missing cleavages. MS/MS spectra were searched with a maximum allowed deviation of 1 Da for the precursor mass and 0.8 Da for fragment masses. Methionine oxidation was selected as a dynamic modification, and the false discovery rate (FDR) was 1%.

For deep PTMs analysis, total proteins from the tumor and adjacent tissues were separated on SDS-PAGE and the ~22 kDa bands corresponding to MNSOD were excised. Proteins in the gel slices were digested with trypsin and peptides were recovered with C18 ZipTip. The extracted and desalted peptides were subjected to peptide fractionation by liquid chromatography on an EASY-nLC 1000 system (Thermo Scientific) equipped with a long C18 column (300 mm × Ø 0.075 mm, 3 μm particles). Samples were fractionated with 120 min linear gradient of 5%–35% acetonitrile/0.1% formic acid at a flow rate of 300 nL/min (solvent A: 0.1% formic acid in water, solvent B: 0.1% formic acid in acetonitrile). The MS and MS/MS spectra were acquired by a LTQ-Orbitrap Elite mass spectrometer (Thermo Scientific) in a data-dependent mode, in which MS/MS fragmentation of the 20 most intense peaks were acquired for every full MS scan. MS/MS spectra were searched against the human protein database using MASCOT and SEQUEST. Trypsin (full cleavage) was specified as cleavage enzyme allowing up to two missing cleavages. MS/MS spectra were searched with a maximum allowed deviation of 10 ppm for the precursor mass and 0.6 Da for fragment masses. The oxidation of various amino acid residues including methionine was selected as dynamic modification, and the false discovery rate (FDR) was 1%. For nitration, tyrosine was selected as dynamic modification. The thresholds for the accepted MS/MS spectra (peptides) were Ions Score of 38 for MASCOT or XCorr of 1.22 × charges for SEQUEST [36]. All modification site assignments were confirmed by manual spectrum interpretation.

For open modification search, a multi-blind spectral alignment algorithm, termed MODification via alignment or MODa [20], was used. Amino acid residues with a delta mass of +16 Da and probability higher than 95% were retained for potential oxidation modifications.

### 4.3. Label-Free Quantification

To quantitatively compare the protein abundances between ccRCC and adjacent tissues, spectral count was performed [37]. This method compares the number of identified MS/MS spectra from the same protein in each of the multiple data sets. For normalization, the spectral counts of each protein were divided by the total spectral counts of all proteins from the same sample. To estimate the levels of oxidation at each oxidized residue, the intensities of all peptide fragments containing the oxidized residue were measured [38] by Progenesis LC-MS software (version 4.1, Nonlinear Dynamics, Newcastle, UK). For quantification of ratio of the oxidized residues, the abundance of oxidized residue was divided by the abundance of unoxidized residue in every four pairs of tissue samples as shown in Table S7.

### 4.4. Pathway and Network Analyses of Dysregulated Proteins in ccRCC

The dysregulated proteins were chosen based on the criteria of *p* < 0.05, presence in every replicate, average spectral counts of >20 and fold change ratio of 1.4 (up or down) in ccRCC comparing with adjacent tissues. For identifying enriched signaling networks and diseases categories, the dysregulated proteins were subjected to bioinformatics tools: STRING (available on: http://string-db.org/) and DAVID functional annotation tool (available on: http://david.abcc.ncifcrf.gov/) [39]. The STRING database version 10 and medium confidence (0.4000) were used and active interaction sources included all default settings, such as text mining, experiments, database, co-expression, neighborhood, gene fusion, co-occurrence. For DAVID functional annotation, DAVID database version 6.8 was used.

### 4.5. Western Blotting (WB) Analysis

Samples were separated on SDS-PAGE gel. After electrophoresis, the proteins were transferred to nitrocellulose (NC) membranes, blocked then probed with primary antibodies against MNSOD (1:750, Santa Cruz, Dallas, TX, USA) and Tubulin (1:10,000, Sigma-Aldrich, St. Louis, MO, USA). Proteins were detected using fluorescence conjugated secondary antibody. The membranes were scanned with Odyssey infrared imaging system (Li-Cor, Lincoln, NE, USA).

### 4.6. MNSOD Enzymatic Activity Assay

Commercial superoxide dismutase (SOD) activity kit (Jiancheng biotechnology, Nanjing, China) was used to measure MNSOD enzymatic activity from kidney tissue lysates according to manufacturer’s instructions and based on modified method as previously reported [16]. Total SOD activity was assayed by the inhibition of xantine/xantine oxidase mediated reduction of cytochrome c, and MNSOD activity was determined with addition of 3 mM KCN to inhibit Cu/ZnSOD.

### 4.7. Data Analysis

The SPSS 17.0 software (SPSS Inc., Chicago, IL, USA) was used for statistical analysis. *p* < 0.05 was considered statistically significant. Statistical analyses between two groups were performed using a Student’s *t*-test, whereas comparisons involving multiple groups were performed with a two-way ANOVA test.

## 5. Conclusions

Our study suggests that MNSOD could be highly susceptible to oxidative modifications in ccRCC. The newly identified oxidative modification sites, particularly those in the C-terminus hydrophobic pocket, may be developed as biomarkers or molecular targets to study ROS regulatory mechanisms, ease the interpretation of previous MNSOD data in tumor biology, and enhance our intervening ability in tumorigenesis [18,19].

## Figures and Tables

**Table 1:** Frequency of +16 modification events of MNSOD at different amino acid residues *.

Amino Acid	A	C	D	E	F	G	H	I	K	L	M	N	P	Q	R	S	T	V	W	Y	Total
Adjacent	3	0	0	0	0	5	0	0	0	0	0	1	0	0	0	0	0	0	28	4	41
Tumor	4	0	0	0	0	22	14	0	0	0	0	12	0	0	0	0	0	2	53	20	127
Total	7	0	0	0	0	27	14	0	0	0	0	13	0	0	0	0	0	2	81	24	168

* Note: total counts of the peptide with a delta mass of +16 Da at the indicated amino acids.

**Table 2:** The modified (oxidized and nitrated) amino acid residues in MNSOD ^a^.

*m*/*z* ^b^	*z*	Peptide with Modification ^c^	Amino Acid Modified	Ions Score (XCorr) ^d^	Expectation Value
877.93	2	**^54^H(ox)HAAYVNNLNVTEEK^68^**	His^54^	80 (4.9)	4.93 × 10^−6^
885.92	2	^54^H(ox)H(ox)AAYVNNLNVTEEK^68^	His^54^, His^55^	44 (3.7)	1.87 × 10^−2^
888.44	2	**^203^AIW(ox)N(ox)VINWENVTER^216^**	Trp^205^, Asn^206 e^	81 (3.8)	4.05 × 10^−6^
888.44	2	**^203^AIWNVIN(ox)W(ox)ENVTER^216^**	Asn^209 e^, Trp^210^	59 (3.9)	5.83 × 10^−4^
1026.02	2	**^135^LTAASVGVQGSGW(ox)GWLGFNK^154^**	Trp^147^	75 (5.4)	1.61 × 10^−5^
1034.02	2	**^135^LTAASVGVQGSGW(ox)GW(ox)LGFNK^154^**	Trp^147^, Trp^149^	68 (4.7)	1.03 × 10^−4^
880.44	2	**^203^AIW(ox)NVINWENVTER^216^**	Trp^205^	66 (4.7)	9.59 × 10^−5^
880.44	2	^203^AIWNVINW(ox)ENVTER^216^	Trp^210^	79 (5.0)	5.04 × 10^−6^
877.93	2	**^54^HHAAY(ox)VNNLNVTEEK^68^**	Tyr^58^	96 (5.6)	1.24 × 10^−7^
892.42	2	^54^HHAAY(no)VNNLNVTEEK^68^	Tyr^58^	27 (3.4)	4.10 × 10^−1^

^a^ The modified peptides in MNSOD (P04179, SODM_HUMAN), which were identified by MASCOT and SEQUEST. See Figure S1 for mass spectra containing b and y ions. ^b^ Precursor mass accuracy <10 ppm. ^c^ Modified peptide sequences. ox: oxidation, no: nitration. The peptide sequences with bold letters indicate novel oxidized/nitrated residues. ^d^ Ions Score (>38) by MASCOT, XCorr (>1.22 × charges) by SEQUEST. ^e^ Asn oxidation combined with Trp oxidation.

## Supplementary Materials

Supplementary materials can be found at www.mdpi.com/1422-0067/18/2/319/s1.
