# Supplementary material for: LC-MS/MS Analysis Unravels Deep Oxidation of Manganese Superoxide Dismutase in Kidney Cancer
Source: Int J Mol Sci. 2017 Feb 4;18(2):319. doi: 10.3390/ijms18020319 (PMC5343855; doi:10.3390/ijms18020319)
Supplement: Supplementary file 1 [file ijms-18-00319-s001.zip › ijms-159250-SupplementaryFigures-Proofreading.pdf]

Supplementary Materials: LC-MS/MS Analysis Unravels Deep Oxidation of Manganese Superoxide Dismutase in Kidney Cancer

Zuohui Zhao, Kazem M. Azadzoi, Han-Pil Choi, Ruirui Jing, Xin Lu, Cuiling Li, Fengqin Wang, Jiaju Lu and Jing-Hua Yang

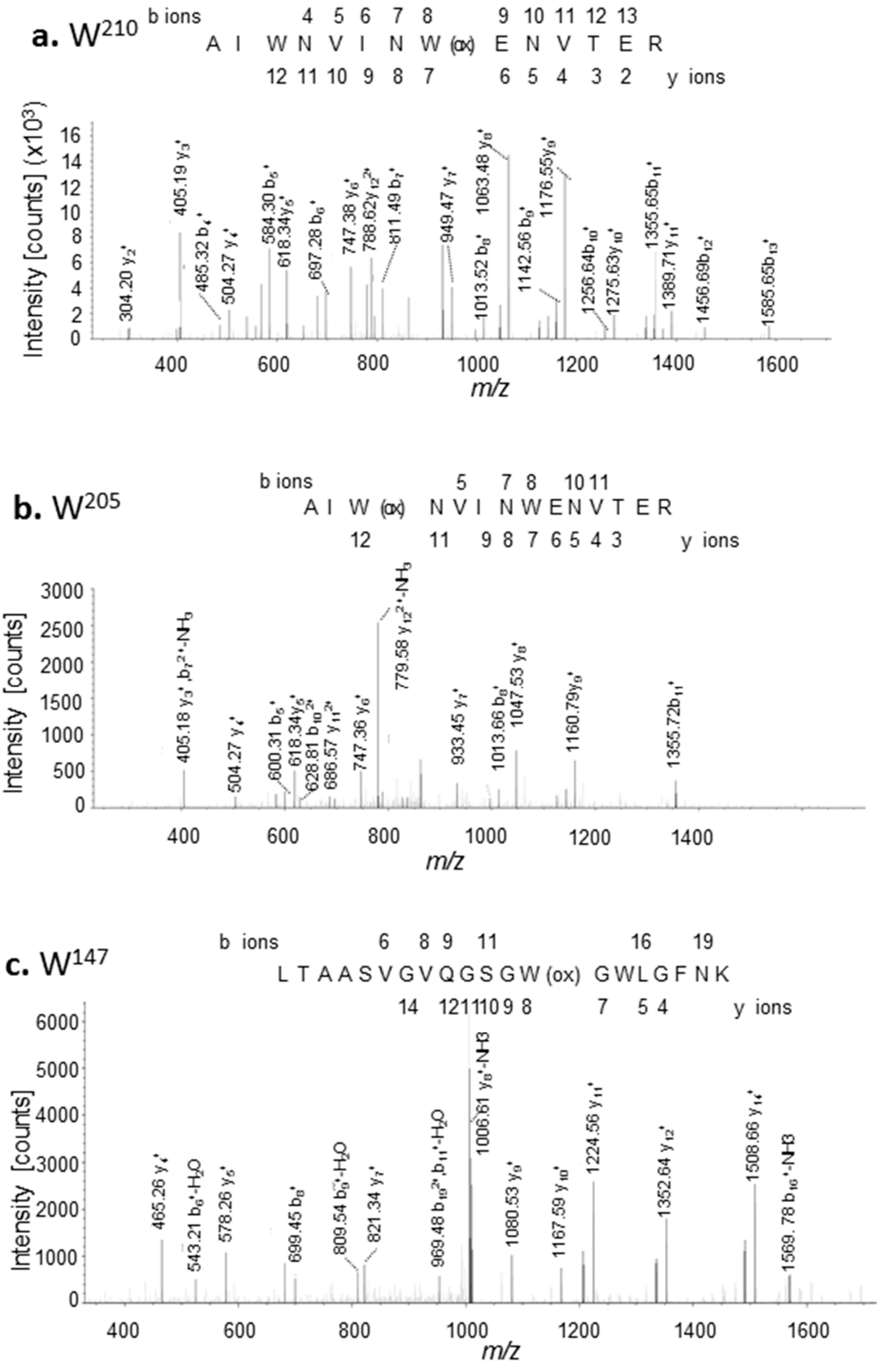

Figure S1. *Cont.*

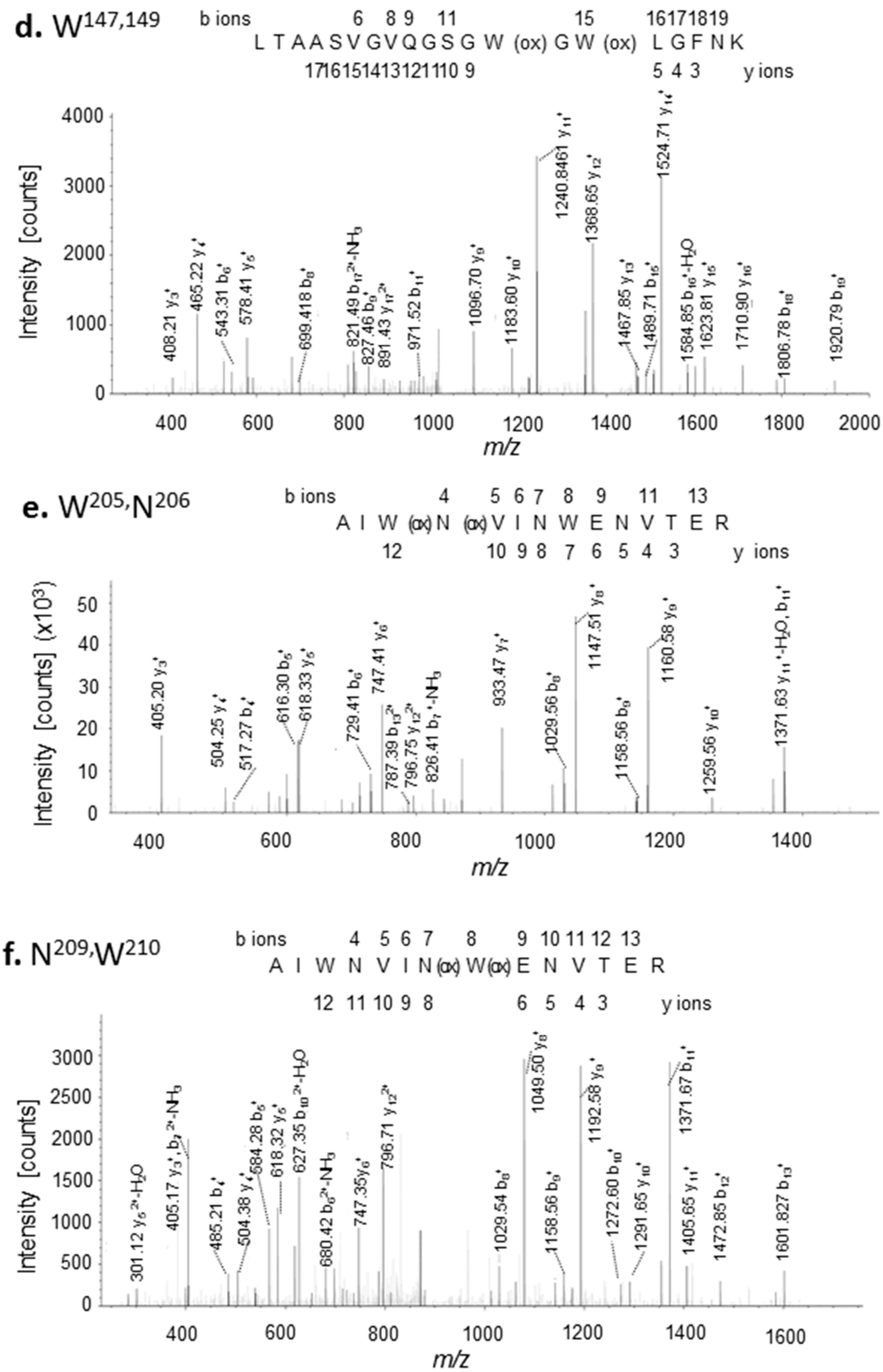

Figure S1. *Cont.*

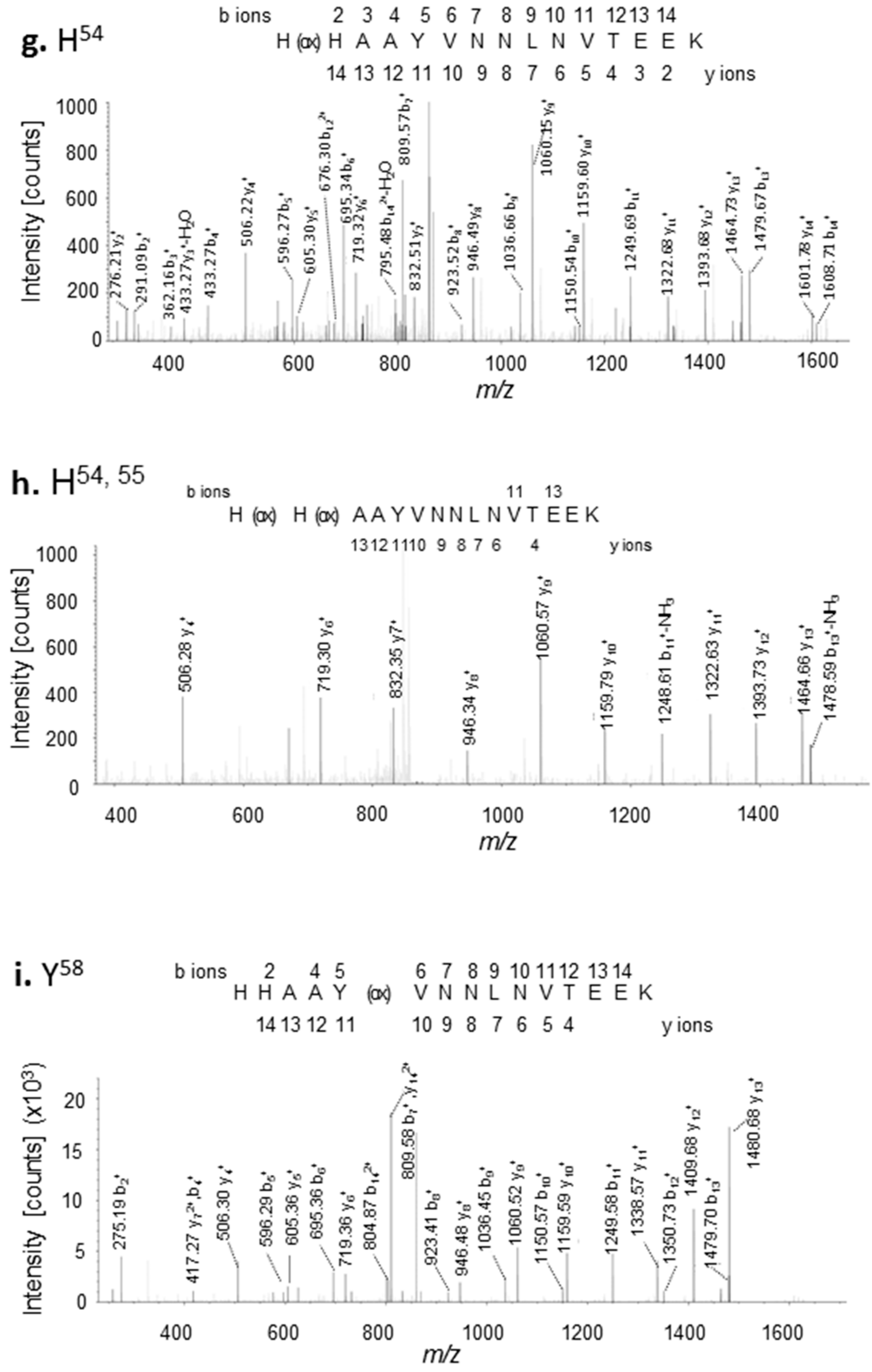

Figure S1. *Cont.*

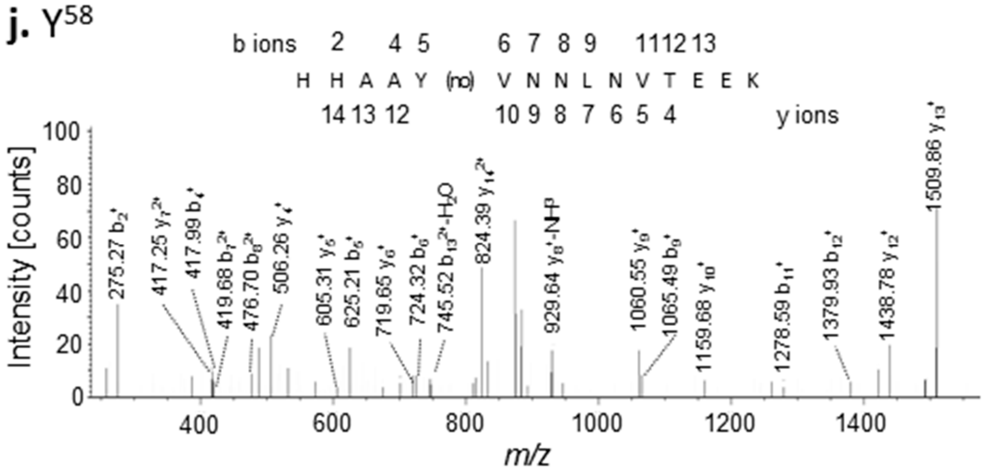

Figure S1. Mass spectra of oxidized/nitrated amino acid residues in MNSOD. The CID spectra of trypsin-digested peptides were searched against human database with a dynamic oxidative modification (+15.99) at some specific amino acid residues or with a dynamic nitrative modification (+44.98) at tyrosine residues. Typical oxidized peptides at W^{210} (**a**); W^{205} (**b**); W^{147} (**c**); $W^{147,149}$ (**d**); W^{205} and N^{206} (**e**); N^{209} and W^{210} (**f**); H^{54} (**g**); $H^{54,55}$ (**h**); Y^{58} (**i**) and typical nitrated peptide at Y^{58} (**j**) are shown. The matched b and y ions are labeled with their mass weights in Dalton. The peptide sequence is shown on the top of each panel, with the matched b and y ions labeled on the top or bottom of the residues, respectively. ox: oxidation, no: nitration.
